# Different but equal: the implausible assumption at the heart of neutral theory

**DOI:** 10.1111/j.1365-2656.2010.01738.x

**Published:** 2010-11

**Authors:** Drew W Purves, Lindsay A Turnbull

**Affiliations:** 1 Computational Ecology and Environmental Science Group, Microsoft Research Cambridge7 JJ Thomson Ave, Cambridge CB3 0FP, UK; 2 Institute of Evolutionary Biology and Environmental Studies, University of ZürichWinterthurerstrasse 190, CH-8057 Zürich, Switzerland

**Keywords:** biodiversity, coexistence, community ecology, density dependence, ecological drift, ecosystem function, life-history manifold, null model, trait variation

## Abstract

**1.**The core assumption of neutral theory is that all individuals in a community have equal fitness regardless of species, and regardless of the species composition of the community. But, real communities consist of species exhibiting large trait differences; hence these differences must be subject to perfect fitness-equalizing trade-offs for neutrality to hold.

**2.**Here we explain that perfect equalizing trade-offs are extremely unlikely to occur in reality, because equality of fitness among species is destroyed by: (i) any deviation in the functional form of the trade-off away from the one special form that gives equal fitness; (ii) spatial or temporal variation in performance; (iii) random species differences in performance.

**3.**In the absence of the density-dependent processes stressed by traditional niche-based community ecology, communities featuring small amounts of (i) or (ii) rapidly lose trait variation, becoming dominated by species with similar traits, and exhibit substantially lower species richness compared to the neutral case. Communities featuring random interspecific variation in traits (iii) lose all but a few fortuitous species.

**4.**Thus neutrality should be viewed, *a priori*, as a highly improbable explanation for the long-term co-occurrence of measurably different species within ecological communities. In contrast, coexistence via niche structure and density dependence, is robust to species differences in baseline fitness, and so remains plausible.

**5.**We conclude that: (i) co-occurring species will typically exhibit substantial differences in baseline fitness even when (imperfect) equalizing trade-offs have been taken into account; (ii) therefore, communities must be strongly niche structured, otherwise they would lose both trait variation and species richness; (iii) nonetheless, even in strongly niche-structured communities, it is possible that the abundance of species with similar traits are at least partially free to drift.

## Introduction

Ecologists are largely agreed that neutral theory has provided valuable null models for community ecology, particularly for species-rich systems (Bell 2001; Chave 2004; Adler, HilleRisLambers & Levine 2007). For example, Hubbell's (2001) formulation of a neutral model has provided an expectation for the dynamics of a plant community lacking any niche structure, or species-specific dynamics or interactions. Such an expectation is clearly valuable as a baseline against which to study the effects of various ecological processes. It is also widely acknowledged that neutral models can reproduce some patterns observed in real communities which had formerly been assumed to result from niche structure and species-specific interactions (Hubbell 2001; Chave 2004; Hubbel 2006). This kind of result helps to identify which patterns contain the most (or least) information about a given ecological process or scientific question (Alonso, Etienne & Mckane 2006; Zilio & Condit 2007). For these reasons, null models based on neutral theory can be expected to remain a useful part of community ecology for the foreseeable future (Clark 2009).

However, the degree to which real ecological communities are actually neutral is a more open question (Gotelli & McGill 2006). That is, what is the distribution of ecological communities along a continuum from purely neutral, through weakly niche-structured, to strongly niche-structured (Adler, HilleRisLambers & Levine 2007)? As well as being of fundamental intellectual interest, the answer to this question is important for predicting how communities might respond to anthropogenic disturbances. For example, the species or trait composition of a community that is more strongly niche-structured will be more stable than that of a neutral community in a static environment. But, it will also show more pronounced, and more predictable, directional responses to environmental change. Moreover, a more strongly niche-structured community will more tightly regulate the biogeochemical functioning of the ecosystem, providing species have differential effects on that functioning (Beare *et al.* 1995; Hector & Bagchi 2007). This in turn implies that the loss of a given species (or type of species) has more impact on biogeochemical functioning in more strongly niche-structured communities.

Naturally, our assessment of how many, and which kind, of communities might occupy different positions along the niche-strength continuum needs to be constrained with data (Etienne & Olff 2005; Gotelli & McGill 2006; McGill, Maurer & Weiser 2006). Long before the recent interest in neutral theory, there was a wealth of empirical evidence that ruled out the key assumption of neutral theory: that all species have equal per-capita growth rates in all situations (see Neutrality vs. ecological drift, below). This assumption is often referred to as the assumption of functional equivalence amongst species (Hubbell 2005). For example, species-habitat correlations (e.g. Whittaker 1956; Walter 1973) and ecological succession (e.g. Cowles 1899), which have been documented in countless communities, are incompatible with the idea that species identity has no implications for per-capita growth rate. More recently, experiments and model-data comparisons have produced results that are incompatible with the assumption of functional equivalence among species. For example, in grassland communities, successful invasion is more likely for species belonging to functional groups that are absent from the resident community (Fargione, Brown & Tilman 2003; Turnbull *et al.* 2005; Petermann *et al.* 2010); and in grasslands and forests the outcome of competition among species can be predicted from measured trait differences (e.g. Pacala *et al.* 1996; Fargione & Tilman 2006; Purves *et al.* 2008). In addition to these direct refutations of the assumptions of neutral theory, there have been a large number of empirical tests of the key predictions of neutral theory (e.g. the distribution of species abundances, species–area relationships: reviewed by McGill, Maurer & Weiser 2006). These tests appear to have rejected neutral theory in most cases (McGill, Maurer & Weiser 2006). However, although this body of empirical evidence appears to rule out pure neutrality, it is much more difficult to interpret in terms of the niche-strength continuum.

Directly assessing the relative importance of niches in maintaining diversity is in fact extremely difficult, requiring detailed field experiments which quantify both baseline fitness differences and the strength of density dependence (but see Levine & Hillerislambers 2009 for an excellent recent example). In contrast, many current tests of neutrality rely on analysing relative abundance patterns, species–area relationships, and/or patterns of how community similarity decays with spatial separation; where the niche signature is likely to be weak (Chave, Muller-Landau & Levin 2002; Purves & Pacala 2005). Given that such tests have little power to place communities on the niche-strength continuum, it is therefore also important to consider, based on first principles, how likely does neutrality seem? Thinking in Bayesian terms (Clark 2004), we could formalize this expectation as a *prior* for the distribution of communities along the niche-strength continuum (Ellison 2004). Outside of a formal Bayesian framework, we can rather ask: if the current empirical evidence does not strongly distinguish between neutrality and niches, do we have any *a priori* reason for preferring one hypothesis over the other?

It has been argued that in the absence of strong evidence for niche structure, the *a priori* preference should be skewed in favour of neutrality, because neutrality is more parsimonious than niche theory (e.g. Hubbell 2005; Hubbel 2006). The argument goes that neutral models are simpler than niche models, because they need to postulate fewer processes, described by fewer parameters. Therefore, in the absence of data, or in the presence of data that does not strongly distinguish between neutrality and niches, we should accept neutrality as the favoured model; a scientific principle known as Occam's razor. But, empirical tests of neutral theory are currently strongly focussed on only a few aspects of ecological communities – for example, relative abundance patterns, species–area curves – and thereby ignore a great deal of other information. We believe that this limited focus has allowed the assumption of neutrality to appear plausible, and even preferable.

In contrast, we argue here from first principles that neutrality is inherently highly implausible, because real communities actually contain species that are observably different in almost every respect. However, neutrality demands that these differences in species’ traits perfectly cancel out, such that the per-capita growth rate of all species, whether small- or large-seeded, fast- or slow-growing, annual or perennial, is identical. We show that such perfect fitness equalization is so unlikely that the *a priori* expectation for the niche-strength continuum should be skewed in favour of niches. The argument laid out here is, in our opinion, sufficient by itself to rule out the possibility of pure neutrality in any ecological community. It also calls into question the robustness of a large body of theoretical results assuming perfect fitness equalization among different species within the same community (e.g. Purves & Pacala 2005; Lin, Zhang & He 2009). In a more general sense, the result implies that, even in the absence of any further empirical tests along the lines of Levine & HilleRisLambers (2009), we can safely conclude that the majority of communities are niche-structured.

## Neutrality vs. ecological drift

To begin, it helps to spell out precisely what a neutral community is. In a neutral community, species identity has no meaning. The fitness of any one individual is independent of its species identity, and independent of the species composition of the community, at all times and in all places. The interaction between any two individuals is also unaffected by their species identities (Chave 2004; Chesson & Rees 2007). This is what is meant by ‘functional equivalence’. Because of this assumption, a neutral community can have no ‘typical’ or equilibrium species composition toward which it returns after disturbance. A neutral community cannot exhibit predictable ecological succession (Sousa 1979; Bergeron 2000), non-random species-habitat correlations (Webb & Peart 2000), stable distributions of species through time and/or space (Clark & McLachlan 2003), or directional changes in species composition in response to perturbations such as climate change (Chapin *et al.* 1995; Iverson & Prasad 1998) or nitrogen deposition (Bobbink, Hornung & Roelofs 1998).

It is also important to distinguish neutrality from the ecological drift of species. Ecological drift implies that the abundances of particular species are poorly regulated, i.e. that they are wholly or partially free to drift upwards and downwards through time (Vellend 2010). This occurs because of stochasticity in births, deaths and the outcome of competition: for example, on average, individuals of a given species might produce 10 000 seeds per year, but in reality the actual number of seeds produced will vary among individuals. Similarly, in lottery-type models with a finite number of sites (such as Hubbell's 2001 model) the choice of exactly which species captures the next vacated site is determined by a random draw. Such stochasticity is necessary in neutral models, as it is the only source of dynamical behaviour, although stochasticity can easily be incorporated into niche-based models as well (Hurtt & Pacala 1995). Thus, the addition of stochasticity does not by itself affect whether the community exhibits exhibit neutrality, stable coexistence, or becomes dominated by one species (Vellend 2010: except under unusual circumstances, for example, when there is a trade-off between the mean and variance of a trait: Lichstein *et al.* 2007).

Intuitively, it might seem that ecological drift of the individual species implies neutrality of the whole community – but this is not the case (Purves & Pacala 2005; Vellend 2010; see Appendix S1, Supporting information for a very simple example). In fact, it is now widely acknowledged that ecological drift of species can occur within communities that are strongly niche-structured (see Hubbel 2006 for an example). In practise, this means that the distribution of species *traits* can be strongly regulated, even where the dynamics of any particular *species* is dominated by ecological drift. For example, a tropical forest may have a typical mix of low wood-density pioneers and high wood-density late-successional species. This mix can be described as regulated if, after the forest is perturbed away from the typical mix, the forest tends to return toward that typical mix. In principle, a forest can behave in this way, even if the dynamics of each particular species is dominated by ecological drift. In contrast, neutrality implies that the community is free to drift from any species composition, to any other species composition. Thus, in a neutral community, there can be no typical mix of traits. A neutral tropical forest could drift to become entirely dominated by pioneers, or entirely dominated by late successionals, just by chance. Thus, although the argument presented here makes an *a priori* case that neutrality is inherently highly improbable, it does not necessarily rule out the ecological drift of particular species.

## Species differences and equalizing trade-offs

It would be easy to believe that real communities were indeed neutral, if real communities were composed of functionally equivalent ‘cryptic’ species, which could only be told apart by sequencing non-functional parts of the genome. In this case, the only process determining the dynamics of species would necessarily be ecological drift, since no ecological process could distinguish between these species. However, to our knowledge, all known communities are composed of species which are neither functionally identical nor cryptic. For example, concentrating on plant communities, we find that co-occurring species are distinguishable morphologically, and exhibit known, qualitative differences in biology, for example nitrogen fixation (yes or no), and seed dispersal mode (wind, animals, both, other). Moreover, quantitative aspects of performance, or quantitative plant traits, typically vary by at least an order of magnitude for co-occurring species: this variation includes, but is not limited to, growth and mortality rates (e.g. Pacala *et al.* 1996; Condit *et al.* 2006), seed size (Westoby, Jurado & Leishman 1992), wood density (Chave *et al.* 2006), height allometry/crown architecture (Poorter, Bongers & Bongers 2006), and leaf characteristics (Wright *et al.* 2004). It appears that wherever the traits of co-occurring plant species have been measured, they have been found to vary substantially among species. Conversations with our zoologically focussed colleagues suggest that substantial trait variation is also a ubiquitous feature of animal communities.

But how can such differences be reconciled with the key requirement of neutral theory, that all species exhibit equal fitness, irrespective of the species composition of the community? Surely substantial trait differences should create substantial fitness differences? The only possible answer is equalizing trade-offs (Chesson 2000a; Turnbull, Rees & Purves 2008; Lin, Zhang & He 2009). An equalizing trade-off is a negative interspecific correlation between two or more traits, which makes interspecific differences in fitness smaller than they would have been otherwise (Chesson 2000a). For example, if more fecund species tend to have shorter life span, then there will be less species-to-species variation in the fitness of species with different fecundities, than there would have been otherwise. Equalizing trade-offs should not be confused with stabilizing trade-offs, which cause density dependence, non-neutral dynamics, the deterministic coexistence of species, and the regulation of the distribution of traits in the community (Chesson 2000a). In other words, stabilizing trade-offs introduce niches. Thus stabilizing trade-offs, which are enabled by species differences and actively equalize species per-capita population growth rates, are not allowed in a neutral community.

Negative correlations among traits of the kind required to enable equalizing trade-offs have been documented in plant communities countless times (e.g. Pacala *et al.* 1996; Dalling & Hubbell 2002; Poorter, Bongers & Bongers 2006); therefore, the idea that communities composed of different species might be neutral, has been deemed plausible (e.g. see Hubbell 2001). This plausibility in turn implies that theoretical studies of neutral communities composed of identical species (as in Hubbell 2001) or species subject to perfect fitness equalizing trade-offs (as in Lin, Zhang & He 2009) are relevant to real communities. However, rarely has it been possible to rule out an alternative explanation: that the observed negative correlations among different aspects of species performances reflect, at least in part, a stabilizing trade-off, i.e. that they have been induced wholly, or in part, by density dependence, such that the shape and magnitude of the trade-offs depends on the species composition of the community.

To understand why equalizing trade-offs are unlikely to enable neutral communities, we first need to remind ourselves that, for a set of species to co-occur for a long time period *without* density dependence, they must have almost exactly equal fitness (Zhou & Zhang 2008). Just as in population genetics, where a small selective advantage for one allele compounds over time and leads to rapid fixation, in population dynamics, a small fitness advantage to one species, or one kind of species, compounds over time, and leads to the rapid exclusion of all other species (Zhou & Zhang 2008). Thus, to enable neutrality, equalizing trade-offs need to be very close to perfect. That is, they need to not just reduce interspecific variation in fitness, but to almost perfectly remove it.

Here, we use simple examples to demonstrate the implausibility of perfect fitness equalizing trade-offs in real communities and show that functional equivalence among species is even harder to achieve than is currently appreciated by most community ecologists – even those who are sceptical about neutral theory. We illustrate our arguments with a simple trade-off between two aspects of performance – life span and annual fecundity – in a plant community, i.e. a space-limited community of sessile organisms. However, the argument we present is general, applying to any trade-off among two or more traits relevant to fitness.

## Life span fecundity trade-off

We consider an idealized community, composed of a number of species *j* = 1 …*n*, where individuals exhibit a species-speific constant annual fecundity *α*_*j*_ (year^−1^), and a constant mortality rate *μ*_*j*_ (year^−1^), throughout their lives. In this case, the expected lifetime fitness of an individual of species *j*, *F*_*j*_, is simply the product of *α*_*j*_ and the expected life span *ρ*_*j*_ (which is equal to 1/*μ*_*j*_), i.e. *F*_*j*_ = (1/*μ*)*α*_*j*_. To simulate the dynamics of this idealized community, we begin with Hubbell's (2001) model, and introduce minimal changes to accommodate variation in mortality and fecundity. The state of the model at any one time is specified by the species identity *j* of the individual occupying each site *q* in the community, which we refer to here as *j*(*q*). The state changes through time as a site *q* is made vacant through random mortality of the individual at *q*, at which point a new species instantly captures *q*, resulting in a new *j*(*q*) value. Thus the dynamics of the system are specified by the mortality probabilities for each site *q*, and by the rule for assigning a vacated site to a species:

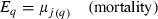
eqn 1.1

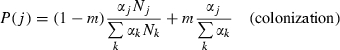
eqn 1.2

where *E*_*q*_ is the annual probability that site *q* will become vacant through mortality; *μ*_*j*(*q*)_ is the annual mortality rate of species *j*; *P*(*j*) is the probability that the newly vacated site will be assigned to species *j*; *N*_*j*_ is the number of sites occupied by species *j* immediately before the mortality event; *α*_*j*_ is the fecundity of species *j*; and the parameter *m* is the probability that the newly vacated site becomes captured via immigration from a regional species pool, rather than from within the local community. In eqn (1.2), the sums over *k* represent sums over all species in the regional species pool. In physical terms, eqn (1) corresponds to assuming: (i) that if the site is captured from within the community, the probability that species *j* captures the next vacated site is equal to the fraction of all of the seeds in the community that are produced by species *j*; (ii) if the site is captured via immigration, all species have equal abundance in the regional pool, and the site is assigned to *j* according to the fraction of seeds arriving from the regional pool that are produced by species *j*. In common with Hubbell's (2001) formulation, eqn (1) implicitly assumes that the number of seeds of each species arriving at each site is equal to the expectation. Therefore, it does not allow for stochasticity in the seed arrival process, which becomes more important as fecundity is reduced. However, in Appendix S2, Supporting information, we show that the results presented here are robust to the inclusion of stochastic seed arrivals, even where fecundities are low (Figs S1 and S2, Supporting information).

## Definition of fitness

It is important to realize that within site-based, lottery-type models of community ecology, such as Hubbell's (2001) model and the variant of Hubbell (2001) used here, the average change in population size, taken over all species, is always zero. This is because there are a fixed number of sites, all of which are filled by a single individual. Thus, any increase in the abundance of one species, must be balanced by a decrease in the abundance of another species. Within this framework, we employ a commonly used measure of fitness which is relevant to the dynamics of the community, i.e. we define the fitness of species *j* as the lifetime output of viable seeds of an average individual of species *j*. When choosing which species captures the next site (eqn 1.2), our model does not distinguish between the seeds of different species; hence according to this definition, a set of species with equal fitness have equal expected per-capita growth rate. Conversely, if there are species with higher fitness according to this definition, those species will capture a disproportionate fraction of newly vacated sites, and so outcompete the other species. Thus, our definition of fitness is sufficient to tell whether or not the community will exhibit neutral dynamics. In alternative models lacking niche structure – for example, where the probability of site capture depends on seed mass rather than seed number, or where the total number of individuals is not fixed – a different measure of fitness would be required. But the same qualitative conclusions would remain, namely, that fitness, appropriately defined, would need to be almost perfectly equal for all species in order for neutral dynamics to occur.

In the neutral case, we can therefore calculate the fitness of species *j* from the traits of species *j*. However, this approach would not be sufficient to understand the dynamics of communities subject to density dependence, where per-capita growth rates depend on both the traits of the species in question, and on how those traits compare with the current mixture of traits present in the community. For example, a pioneer tree species would have greater per-capita growth rate in a landscape currently dominated by late-successional trees, than in a landscape currently dominated by other pioneers. In such niche-regulated communities the concept of fitness can become difficult and needs to be applied with care (Chesson 2000a; Adler, HilleRisLambers & Levine 2007; Levine & HilleRisLambers 2009).

## A perfect trade-off

Returning to our lottery model lacking density dependence, it is a simple matter to derive the functional form of the equalizing trade-off between mortality rate and fecundity that, if it were exhibited in reality, would lead to all species having equal lifetime fitness:

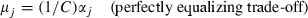
eqn 2

**Fig. 1:**
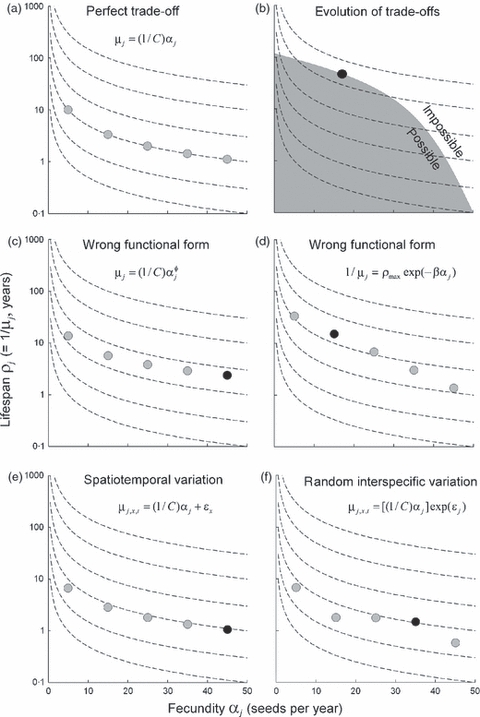
Negative trade-offs do not imply equal fitness. Each panel shows a set of equal fitness isoclines (dashed lines). Any two combinations of life span and fecundity that lie along the same isocline, confer the same expected lifetime fitness. Panels (a), and (c–f), each show a putative community of five species (circles) following a negative trade-off between life span and fecundity following the given equation. If, and only if, the negative trade-off happens to perfectly follow the shape of an equal fitness isocline (a), do the species have equal fitness such that they can co-occur for long periods in the absence of niche structure and density dependence. In all other cases (c–f) one species will be fitter than the others (shown in black). Panel (b) shows why negative trade-offs are not expected to follow the shape of an equal fitness isocline. The shape of the isoclines is set by the ‘top down’ requirement for equal fitness – in this case, the requirement that the product of life span and annual fecundity be the same for each species. In contrast, the shape of the trade-off is determined by quite separate factors, namely, various ecological and biophysical constraints that delineate possible combinations of traits (shown in grey) from impossible combinations. Fitness is increased by an increase in life span, an increase in fecundity, or both, and so species are expected to evolve toward the edge of the region of possible trait combinations; i.e. to evolve the greatest life span for a given fecundity. Without density dependence, one combination of traits along this edge is expected to confer superior fitness compared to all other combinations (black circle in panel b).

**Fig. 2:**
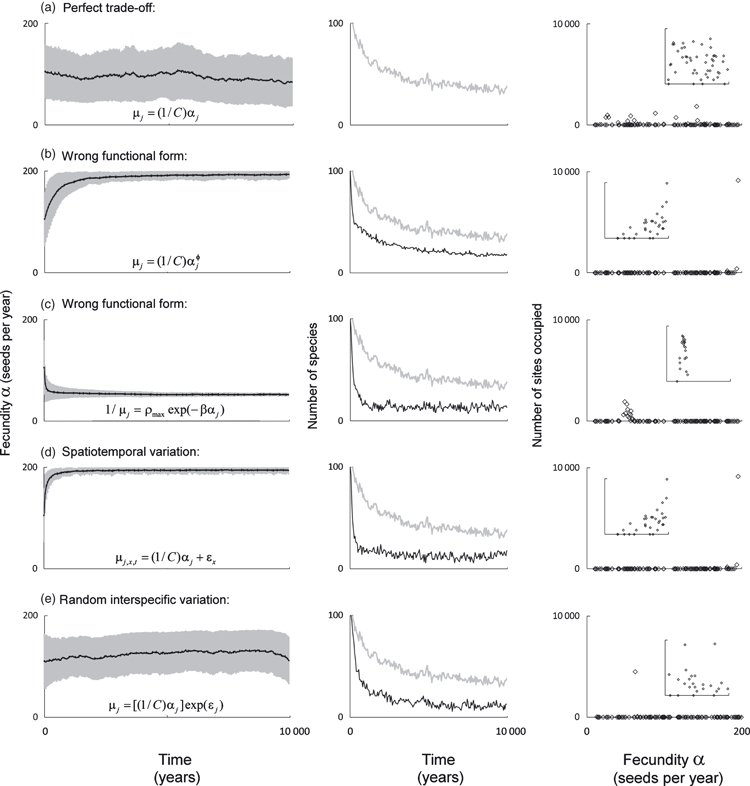
Simulations of population dynamics within space-limited communities lacking any form of niche structure or density dependence. The model used for simulations is very similar to Hubbell's (2001) neutral model (see text). Within each community, life span is negatively correlated with annual fecundity according to the equation given with the left panel (see main text). Left panels: dynamics of mean fecundity α (dark line + grey region gives mean ± 1 standard deviation). Middle panels: dynamics of species richness (black) vs. the dynamics from the truly neutral case (grey). Right panels: state of the community at the end of the simulation, each symbol showing one species. Insets show the same information on a logarithmic vertical axis. As the results show, except in the special case of a perfect trade-off with no spatiotemporal variation and no random interspecific effects (a), trait diversity collapses (b–e, right panels) and species richness is much lower than in the neutral case (b–e middle panels). Simulation results for rapidly varying temporal variation following eqn (6) (not shown) were extremely similar to those for spatial variation following eqn (5) (d).

Where *C* is the lifetime fitness shared by all species. This equal fitness in turn implies neutral dynamics. One way to visualize this is to plot *μ*_*j*_ vs. *α*_*j*_ for different values of *C* (Fig. 1). This provides a set of ‘equal fitness isoclines’, where each isocline corresponds to a set of combinations of *ρ*_*j*_ and *α*_*j*_ that confer equal fitness. Now consider two species *j* and *k*. In a neutral community, *j* and *k* can co-occur for a long period of time if and only if they have equal lifetime fitness, i.e., they are both on the same equal fitness isocline. Otherwise, one species will quickly drive the others to extinction. The argument extends to a multi-species community: any set of species *j* = 1 …*n* can co-occur for long periods if and only if all species lie along the same equal fitness isocline. This is the signature of a perfectly equalizing trade-off. As expected, simulations of this community show pure ecological drift of particular species and, more importantly, pure drift of the distribution of species traits (Fig. 2a).

## Improbability

The problem with this argument is there is no biological or ecological reason why a given set of species should happen to lie on an equal fitness isocline. To generate a neutral community in this case, we deliberately derived the functional form of the trade-off between mortality and fecundity (eqn 2) in order to achieve the end result that species would have equal fitness. We wanted the community to exhibit neutral dynamics, and so we solved for a relationship between life span and fecundity (eqn 2) that would make this true. Crucially, we have provided no biological reasoning, or empirical evidence, supporting the idea that the functional form relating life span and fecundity follows the shape of an equal fitness isocline.

This approach begs the question – why should the trade-off follow the shape of an equal fitness isocline, rather than some other shape? In reality, trade-offs between different aspects of performance will be determined by a variety of processes, but primarily by constraints on different aspects of performance imposed by biophysics and ecology (Fig. 1b). These constraints delineate combinations of different traits and aspects of performance that are possible, from those that are not. Species are then expected to evolve toward the edge of the constraint surface, at which point this edge defines a life-history trade-off (Fig. 1b).

To illustrate, consider the evolution of a plant species, concentrating on just two aspects of performance–life span and annual fecundity – while holding all other aspects constant (e.g. growth rate, allocation to vegetative reproduction, etc.). For this species, life span might be increased by a larger root system (reducing the risk of drought death), thicker leaves (reducing both drought risk and herbivore damage), a larger carbon store (that can be drawn upon in times of reduced carbon fixation or used to replace lost tissues), or an increased concentration of protective compounds (reducing herbivore damage). Similarly, annual fecundity might be increased by more flowers, more ovules per flower, or larger or more nectar-rich flowers (to attract pollinators more efficiently). Crucially, each of these physical features comes at a cost to the plant in terms of resources (e.g. carbon, nitrogen) such that a given unit of resources allocated to a given feature, cannot be allocated to another feature. Thus, considering all features together, a plant with a finite reserve of resources can achieve some combinations of features, and not others. The constraints on the combinations of physical features that are possible, then translate into a constraint on which combinations of performance are possible, as illustrated in Fig. 1b. To understand why plants should evolve toward the edge of this constraint surface, we need only note that an increase in any one aspect of performance, with others held constant, increases fitness, and hence is favoured by natural selection. Thus, natural selection will tend to make species evolve such that they express combinations of different aspects of performance that are on the edge of what is possible.

This allocation argument explains why equalizing trade-offs – negative correlations among different aspects of performance – are expected to be common in nature. However, it also illustrates why *perfectly* equalizing should be extremely rare. First, a unit of resource allocated to one feature may have much less effect than the same unit of resource allocated to an alternative feature that affects a different aspect of performance. For example, a small amount of extra carbon allocated to flowers may have a large affect on fecundity, whereas the same amount of carbon allocated to roots may have a small effect on survival. Secondly, most allocation decisions will affect more than one aspect of performance. For example, increased allocation to stem might provide support structure for more leaves (increasing growth rate), more flowers (increasing fecundity) and hold the leaves and flowers at a great height (increasing both growth rate and pollination success).Thus, we expect the edge of the constraint surface to have a complex, nonlinear shape determined primarily by exactly how allocation to different physical features affects different aspects of performance.

Viewed in this way, it becomes clear that it is extremely unlikely that the wide variety of biophysical and ecological constraints on the evolution of species will lead to a life-history trade-off that happens to place co-occurring species along an equal fitness isoclines – even if the biophysical constraints cause a strong negative correlation among two or more traits. As Fig. 1 shows, a negative correlation alone is not sufficient to confer equal fitness, because many possible negative correlations nonetheless do not conform to an equal fitness isocline. Rather, neutrality requires that *all fitness-relevant traits happen to be negatively correlated in exactly the way required to confer equal fitness on all co-occurring species*. To illustrate, we return to the idealized neutral community described above and introduce a series of simple changes to eqn (2), each of which makes the trade-off between life span and fecundity imperfect. We show that each of these changes destroys the equality of fitness among species and hence destroys the neutrality of the community.

First, and most importantly, *any change in the functional form* of the relationship between mortality and fecundity away from that required for equal fitness, means that the species cannot lie along an equal fitness isocline. A minor change to eqn (1) is given by introducing an exponent:

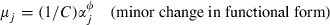
eqn 3

Providing *φ* > 0, this new equation still describes a perfect, negative correlation between fecundity and life span (Fig. 1c). But, this new equation gives equal fitness among species for *φ* = 1 only. Under any other value of *φ*, lifetime fitness is now a function of fecundity: 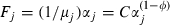. Simulations of the dynamics of a community structured according to eqn (3), give dominance by a single or a few species with very similar fecundity, with very rapid exclusion of all other species (Fig. 2). Depending on the value of the exponent *φ*, the dominant species are either those with the greatest fecundity (if 0 < *φ* < 1) or the greatest life span (if *φ* > 1).

In eqn (2), the functional form of the relationship between fecundity and mortality was at least chosen to be close to that required for equal fitness, differing only by an exponent (eqn 2 vs. 3). But there is no reason to expect that these two functions (the trade-off, and the equal fitness isocline) should be related at all (see Fig. 1b). For example, eqn (3) is unrealistic because it gives plants with zero fecundity a zero mortality rate, and hence an infinite life span. This problem can be avoided by using a more plausible functional form where, as fecundity approaches zero, life span approaches a maximum value *ρ*_max_ (year):

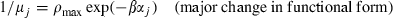
eqn 4

Under this functional form, there is no combination of *ρ*_max_ and *β* that confers equal fitness on all species, because fitness is a function of fecundity for all values of *ρ*_max_ and *β*, i.e. *F*_*j*_ = *ρ*_max_*α*_*j*_ exp (−*βα*_*j*_). As expected, simulations of the dynamics of this community give dominance by one or a few species with very similar fecundities – but this time, the dominant species have an intermediate fecundity (Fig. 2). Again, these results occur despite the fact that fecundity and life span are perfectly negatively correlated (eqn 4, Fig. 1d).

More generally still, we can imagine the universe of all possible functions describing a perfect negative relationship between life span and fecundity. This universe is very large, including (for example) various nonlinear, sigmoid and threshold-like functions. Given the variety of biophysical and ecological constraints to which species are subject, we would expect to find a very wide variety of these functional forms represented in real communities. But within this extremely large universe, there is exactly one function that results in equal fitness, and neutral dynamics. In this way, we can visualize the prior probability of neutrality: it is the probability of selecting, at random, that one special functional form from the extremely large universe of all possible functional forms.

## Fragility to spatiotemporal variation and random species differences

The argument above explains why perfectly fitness-equalizing trade-offs are unlikely to occur in reality. In this section, we show that, even if such a trade-off did occur, it could easily be destroyed by other factors.

The first of these factors is *spatial and temporal environmental variation in performance*. To give neutrality the best chance of occurring in the face of this variation, we return to the perfect fitness-equalizing functional form (eqn 2) despite that fact that this functional form is unlikely to occur in reality. We then introduce spatial variation in performance, or temporal variation, as follows:

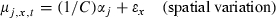
eqn 5

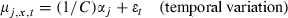
eqn 6
for a set of communities in different locations *x*, and measured at different times *t*; where *ɛ*_*x*_ and *ɛ*_*t*_ are random effects on mortality, associated with our particular local community *x*, or associated with time *t* (note that to prevent mortality rates becoming negative we constrained *ɛ*_*x*_ ≥ 0 and *ɛ*_*t*_ ≥ 0, which in turn implies that (1/*C*)*α*_*j*_ is the minimum possible mortality rate). Crucially, in eqns (5, 6) these effects occur in such a way that they act equally on all species, regardless of species identity, mortality rate or fecundity. That is, for spatial variation, within any particular local community, all species are subject to the same *ɛ*_*x*_; and for temporal variation, at any particular time *t,* all species are subject to the same *ɛ*_*t*_.

Despite this lack of species specificity, in the presence of spatial variation, fitness within our local community *x* now varies among species (Fig. 2). That is, as long as *ɛ*_*x*_ is non-zero, fitness is now a function of fecundity: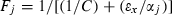. Again, this variation in fitness occurs despite the fact that, whatever the value of *ɛ*_*x*_, life span and fecundity are perfectly negatively correlated (Fig. 1e). As expected, simulations show that such communities become dominated by a small number of species with similar traits (Fig. 2d, right panels). The winning species are those with the combination of mortality and fecundity closest to the optimal combination, given the value of *ɛ*_*x*_ for that location. With the background trade-off between life span and fecundity following eqn (2) (as shown in eqns 5, 6), the winning species are those with the greatest fecundity. In the presence of different functional forms (not shown) the winning species could have intermediate fecundities.

Note, however, that the above treatment of spatial variation, and the simulation results presented in Fig. 1, consider only a single local community *x*, subject to one spatial effect, *ɛ*_*x*_. Alternatively, we could consider a metacommunity, composed of multiple local communities subject to multiple *ɛ*_*x*_ values. In this case, we would expect each local community *x* to become dominated by those species with the optimal combinations of traits, given *ɛ*_*x*_. If the optimal trait combinations differed from community to community, the result at the metacommunity scale would be deterministic coexistence via the spatial storage effect (Chesson 2000b). The trait variation and species richness of the metacommunity would not collapse. However, neutrality would have still been destroyed, because the trait composition would no longer be free to drift within any local community, or at the metacommunity scale.

The effects of temporal variation are similar to those of spatial variation. In the presence of temporal variation, at any given time *t* fitness is a function of fecundity such that one set of trait combinations confers greater fitness than any other combinations. Once again, with the background trade-off between life span and fecundity following eqn (2) (as shown in eqns 5, 6) the winning species are those with the greatest fecundity, whereas with a different functional form (not shown) they could have intermediate fecundities. Thus, at any one time, the community exhibits non-neutral dynamics. If the value of *ɛ*_*t*_ remains unchanged for very long periods, there is sufficient time for the community to become dominated by those species with traits closest to that optimum. In contrast, if *ɛ*_*t*_ varies from one time to the next, the identity of the most fit species could vary through time. This did not occur in the simulations carried out here, because the background trade-off follows eqn (1) where the fittest species is always the one with the greatest fecundity. But it could occur with a different functional form (not shown). In this case, the community at any one time would be moving toward dominance by one species, but the identity of this species would be changing through time. Nonetheless, over the long term a particular regime of temporal variation will tend to favour particular combinations of traits above others, and thus destroy neutrality. Simulations of communities subject to rapid temporal variation (not shown) confirm this expectation, exhibiting a rapid loss of trait diversity and reduced species richness compared to the neutral case.

Now, consider *random differences in the performance of species*. Again, to give neutrality the best chance of occurring in the face of this variation, we set the functional form relating life span and fecundity to eqn (2). We then impose:

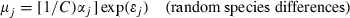
eqn 7
where *ɛ*_*j*_ is a random species effect drawn from a distribution with mean 0, irrespective of the fecundity or mortality rate of *j*. It is relatively obvious that such differences destroy neutrality (Fig. 1): a set of species could co-occur for a long period only if they happened to have all received *ɛ*_*j*_ = 0, or happen to have received a set of *ɛ*_*j*_ values that shifted them onto the same equal fitness isocline. By far the most likely outcome of eqn (7) is that one or a very few species end up with fitness sufficiently greater than the other species, that they rapidly exclude all other species (Fig. 1f). Note once again that this result occurs despite the fact that life span and fecundity are strongly and negatively correlated (although the correlation is no longer perfect: Fig. 1).

Random species differences also differ from alternative functional forms (eqns 3, 4) and spatiotemporal variation (eqns 5, 6) in that random species differences destroy the continuity of the set of species fitness. Specifically, in the presence of alternative functional forms and spatiotemporal variation as employed here, two species with extremely similar traits necessarily have extremely similar fitness. As such, it can take a long time for the species with greatest fitness to drive similar species extinct (see the discussion of continuity in Purves & Pacala 2005). In contrast, in the presence of random species differences, a pair of species with very similar traits will tend to exhibit dissimilar fitness, simply because they will tend to have received different random species effects. This explains why the loss of species richness is more rapid under random species differences (Fig. 1e middle panels) compared to the other cases (Fig. 1b–d middle panels).

Finally, note that these four processes – variation in the functional form of the trade-off, spatial and temporal variation in performance, and random performance differences – are not mutually exclusive. As each of these features is introduced into a community, equality of fitness among species becomes progressively harder to achieve. This is important because it is possible to find some special mathematical formulations of some of the above processes that, in isolation, do not destroy equal fitness (e.g. spatial environmental effects that act multiplicatively, rather than additively, on annual mortality rate). We note, however, that in reality it seems extremely unlikely that that the formulations governing these individual processes should happen to be the special cases that result in equal fitness among species.

## Discussion

To summarize our argument for the improbability of neutrality: (i) co-occurring species exhibit a wide variety of trait and performance differences; (ii) neutrality requires equalizing trade-offs that cancel out those differences to leave identical fitness for all species; (iii) such perfectly fitness equalizing trade-offs are highly improbable, and highly fragile. Returning to our Bayesian analogy (see Introduction), we can now combine a low prior for neutrality, with the large body of data that rules out pure (or nearly pure) neutrality (ecological succession, species-habitat correlations, non-random correlations among species in space and time), that directly refutes the assumption of functional equivalence (e.g. Fargione, Brown & Tilman 2003), that rejects the predictions of neutral theory (reviewed by McGill, Maurer & Weiser 2006) or that rules in strong niche regulation (e.g. Levine & HilleRisLambers 2009). In our opinion, all of this leaves us with a very low posterior probability for neutrality as the explanation for the long-term co-occurrence of contrasting species within ecological communities.

In contrast, niche theory has identified a limited number of spatio-temporal coexistence mechanisms that allow the deterministic coexistence of large numbers of species (Chesson 2000b), as well as many ecological processes that can underlie these mechanisms (e.g. habitat specialization, variation in germination requirements, Janzen-Connell effects, etc.). All these mechanisms induce density dependence, which causes the abundance of the species remaining in the community to become adjusted until each species has equal per-capita growth rate. If the species composition is perturbed, density dependence immediately induces species differences in per-capita growth rates, which causes the species composition to return toward the pre-perturbed state. Thus, it is crucial to distinguish between the predictions of niche theory, which states that species are expected to exhibit equal per-capita population growth rates *when and only when the species composition of the community is at its equilibrium state*; from the predictions of neutral theory, which states that species are expected to exhibit equal per-capita growth rate *regardless of the species composition of the community*.

Importantly, coexistence via density dependence is robust to species differences in baseline fitness (i.e. fitness measured within some reference community, e.g. an empty landscape, or a community where all species are equally represented). If fitness differences are not too large, species with lower baselines fitness can remain in the community because their reduced abundance results in reduced density dependence, thus allowing them to achieve a per-capita growth rate that is equal to that of the more common species (Chesson 2000a). Thus, coexistence under niche structure is robust to the same ecological realities discussed above – arbitrary nonlinear functions relating different traits, spatial and temporal environmental variation, idiosyncratic species differences – that destroy neutrality.

## Returning to the continuum

The results presented above effectively rule out the possibility of purely neutral dynamics in any community that exhibits large trait differences. That is, they rule out the possibility that the distribution of traits is free to drift within any community. But they also help to constrain our understanding of the likely relative strength of neutrality vs. niches in structuring communities in general, by altering our perception of the likely magnitude of interspecific differences in baseline fitness. Without equalizing trade-offs, the observed variation in multiple species traits imply fitness differences of several orders of magnitude. Equalizing trade-offs can be expected to reduce this fitness variation. But, as explained above, this compensation can be expected to be far from perfect in most cases. Thus, we expect substantial differences in baseline fitness to remain even after equalizing trade-offs have been accounted for. Species with very different baseline differences in fitness can only be maintained in a community via strong niche regulation. This, in turn, implies that the distribution of traits in most communities is strongly regulated – i.e. that most communities are far from neutral.

## Supporting Information

**Appendix S1.** Neutrality vs. ecological drift.

**Appendix S2.** Simulations.

**Fig. S1.** 10 000-year simulations of space-limited communities lacking any form of niche structure or density dependence.

**Fig. S2.** Simulations of communities with increasing levels of stochasticity in seed arrivals.

As a service to our authors and readers, this journal provides supporting information supplied by the authors. Such materials may be re-organized for online delivery, but are not copy-edited or typeset. Technical support issues arising from supporting information (other thanmissing files) should be addressed to the authors.
