# Supplementary material for: Different but equal: the implausible assumption at the heart of neutral theory
Source: J Anim Ecol. 2010 Nov;79(6):1215–25. doi: 10.1111/j.1365-2656.2010.01738.x (PMC3025117; doi:10.1111/j.1365-2656.2010.01738.x)
Supplement: Supplementary file 1 [file jane0079-1215-SD1.doc]

**Supporting information for Purves & Turnbull: ‘Different but equal: the implausible assumption at the heart of neutral theory’**

**Appendix S1: neutrality vs ecological drift**

As outlined in the text (neutrality vs ecological drift) and discussed at length in Purves & Pacala (2005), the ecological drift of the abundance of individual species, can occur within communities that are highly niche-structured. Intuitively, it seems surprising that, if each species in a community is free to drift, the species composition can be highly regulated. Therefore, to complement the examples given in the main text, and in Purves & Pacala (2005), we here provide a very simple mathematical analogy to a niche-structured community featuring ecological drift.

Imagine a community of total abundance 100 (i.e. comprising 100 sites each occupiable by one individual), made up of four species *a, b, c, d,* and comprising two guilds with two species within each guild (*a* and *b* in one guild: *c* and *d* in the other). Assume that the proportion of the community dominated by each guild is very strongly regulated at 50 sites to each guild. Then we know that at any one time: $N_{a}+N_{b}=50$ (A1.1)

$N_{c}+N_{d}=50$ (A1.2)

Now, within this community we can select any abundance between 0-50 for species *a*, and then find an abundance for species *b* that satisfies Eq. A1.1. Similarly, we can select any abundance between 0-50 for species *b*, and find the abundance for species *a* that satisfies Eq. A1.1. And so on for species *c* and *d*. Thus, our description of this community allows for the dynamics of each component species to be dominated by drift. But, the mixture of the two guilds in the community is perfectly regulated at 50:50, i.e., it is not free to drift at all. This in turn implies that the distribution of the *traits* that place a species into either guild (e.g. nitrogen fixation or not) is itself perfectly regulated, despite the drift of individual species. Importantly, strong regulation of traits, and ecological drift of species, can also occur where species differences are continuous i.e., where species cannot be assigned to discreet guilds (Purves & Pacala 2005); and can occur where the niche regulation is weaker than in the example given here.

**Appendix S2: simulations**

We carried out simulations of a lottery model similar to Hubbell’s (2001) model, but with species-specific variation in mortality and fecundity following the equations given in the main text (Eq. 1 – 6). The state of the model at any one time is specified by the species identity *j* of the individual occupying each site *q* in the community, which we refer to here as$j(q)$. The state changes through time as a site *q* is made vacant through random mortality of the individual at *q* , at which point a new species instantly captures *q*, resulting in a new$j(q)$ value. Thus the dynamics of the system are specified by the mortality probabilities for each site *q*, and by the rule for assigning a vacated site to a species:

$E_{q}=\mu_{j(q)}$ [mortality] (A2.1)

$P(j)=(1-m)\frac{\alpha_{j}N_{j}}{\sum_{k} \alpha_{k}N_{k}} + m\frac{\alpha_{j}}{\sum_{k} \alpha_{k}}$ [colonization] (A2.2)

Where $E_{q}$ is the annual probability that site *q* will become vacant through mortality; $\mu_{j(q)}$ is the annual mortality rate of species *j*; $P(j)$ is the probability that the newly-vacated site will be assigned to species *j*; $N_{j}$ is the number of sites occupied by species *j* immediately before the mortality event; $\alpha_{j}$is the fecundity of species *j*; and the parameter $m$ is the probability that the newly-vacated site becomes captured via immigration from a regional species pool, rather than from within the local community.

In physical terms, Eq. A2.2 corresponds to assuming (1) that if the site is captured from within the community, that the probability that species *j* captures the next vacated site, is equal to the fraction of all of the seeds in the community, that are of species *j*; (2) if the site is captured via immigration, all species have equal abundance in the regional pool, and the site is assigned to *j* according to the fraction of seeds coming in from the regional pool, that are of species *j*.

To implement the simulations, we employed the Gillespie algorithm, which allows for: (1) the efficient simulation of models governed by discrete stochastic events; (2) the correct assignment of time as the model is simulated. We assumed a total community size of 10000 and set $m$ = 0.0010. We generated a species pool of 100 species, and assigned each species a parameter $\alpha_{j}$ at random, by drawing numbers from a uniform distribution of 5-200. For each species *j*, we then assigned a value of $\mu_{j}$, using one of Eqs 1 – 6 given in the main text. For Eqs. 1 and 2, we used *C* = 1000. Eq. 3 (minor change in functional form) we used $\varphi$ = 0.80. For Eq. 4 (major change in functional form) we used $\rho_{\max}$ = 200.0 and $\beta$ = 0.020. When using Eq. 5 (spatial variation), we selected a single value of $\varepsilon_{x}$ = 0.050 to illustrate how spatial variation in performance can destroy an otherwise perfect trade-off. This $\varepsilon_{x}$ value applied for the remainder of the simulation. Note that this approach implies that the whole community is a single location ‘x’. The effects of spatial variation in performance, on a collection of locations, would be to cause deterministic coexistence via the ‘spatial storage effect’ (Chesson 2000). When using Eq. 6 (temporal variation), we used the same procedure to select $\varepsilon_{t}$values. Doing this once for the simulation, corresponding to temporal effects that vary very slowly, would necessarily have given identical results to spatial variation, and so these simulations were not carried out. However, we did simulate the effects of rapidly-varying $\varepsilon_{t}$ values, by re-selecting the $\varepsilon_{t}$ , from a uniform distribution between 0 and 0.10, whenever the time passed a whole year (i.e. immediately after 1.0 years, 2.0 years, and so on). When using Eq. 7 (species differences), we selected a single value of $\varepsilon_{j}$ for each species *j*. The $\varepsilon_{j}$ values, which we drew from a uniform distribution between -0.10 and +0.10, applied for the remainder of the simulation. Note that in all cases, we chose parameter values that represented relatively small deviations from the perfect trade-off (e.g. $\varphi$ = 0.80 in Eq. 3), i.e., we did not choose unrealistic parameter values in order to cause dramatic differences in fitness among species.

Throughout the simulations, we recorded the mean and standard deviation of fecundity$\alpha$ over all sites *q* (see Fig. 2):

$\alpha_{mean}=(1/M)\sum_{q=1}^{M} (\alpha_{j(q)})$ (A2.3)

$\alpha_{sdev}=(1/M^{2})\sum_{q=1}^{M} [(\alpha_{j(q)})-\alpha_{mean}]^{2}$ (A2.4)

Where *M* is the total number of sites. To initialize the model, we assigned each site *q* to one species drawn randomly from the list of 100 species regardless of fecundity or lifespan. We then simulated dynamics for a subsequent 10,000 years, recording the state of the community every 50 years.

**Stochasticity in seed arrivals**

The formulation for site capture given above (Eq. A2.2) and repeated in the main text (Eq. 1.2) does not allow for stochasticity in seed arrival at newly vacated sites. Rather, the formulation implicitly assumes that the number of seeds arriving at the site from each species is exactly equal to the expected number, for every site, each time it is vacated. Whereas, in reality, the actual number of seeds arriving at each vacated site, can vary due to the stochasticity of the seed arrival process. Within some model formulations of lottery models, this stochasticity can allow inferior species to sometimes capture sites by ‘forfeit’ simply because no other seed arrived at that site, which in turn can slow exclusion (Hurtt & Pacala 1995; Hubbell 2006). Because of this, we carried out simulations to test whether the rapid collapse of trait diversity seen when using the expectation for seed arrivals (Eq. A2.2), was also seen when using a more realistic formulation with stochastic seed arrivals. We implemented stochastic seed arrivals in the simplest way possible, by breaking Eq. A2.2 into two parts, representing respectively seed arrivals, and competition for the new site:

$P(j)=(1-m)\frac{S(j)}{\sum_{k} S(k)} + m\frac{\alpha_{j}}{\sum_{k} \alpha_{k}}$ (A3.1)

$S(j)=\text{Poisson}\{\alpha_{j}N_{j}\}$ (A3.2)

Where Poisson{} represents a random number drawn from a Poisson distribution with mean as given in parentheses. Note that we still use the expectation for site capture by immigrant seeds (second term on the right hand side in Eq. A3.1).

Fig. S1 shows the results of introducing stochastic seed arrivals into the simulation results given in Fig. 2. As Fig. S1 shows, the stochastic seed arrivals slow competitive exclusion slightly, but otherwise have no material effect on the results. However, the impact of stochastic seed arrivals is known to be greater, when the expected number of seeds arriving at a site is smaller (Hurtt & Pacala 1996; Hubbell 2006). Could the results presented in Fig. S1 be substantial overestimates of the rate of exclusion, because the numbers of seeds used were unrealistically high? We think this is not the case, for two reasons. First, our average annual fecundity values are in the range 5-200 seeds per year which, when put through the equations for site capture (e.g. Eq. 3.2) implies that, on average, each vacated site has a total of 5-200 seedlings present in the site from which to fill the site. This is quite low in comparison to (for example) the gaps in closed canopy tropical forests studied by Dalling & Hubbell (2002) which contained 4000 – 10000 seedlings (see also Turnbull *et al.* 2009). Second, we carried out a second set of simulations, reducing the effective fecundity of each species by a factor of 10 or 100:

$S(j)=\text{Poisson}\{[\alpha_{j}N_{j}]/10\}$ (A4.1)

$S(j)=\text{Poisson}\{[\alpha_{j}N_{j}]/100\}$ (A4.2)

Giving an average total seed arrival to each site of only 0.5-20 seeds or 0.05-2 seeds respectively. These are low numbers for any conceivable space-limited plant community. Even with these reductions, stochasticity of seed arrival had no material effect on the collapse of trait variation and species richness in this case (Fig. S2).


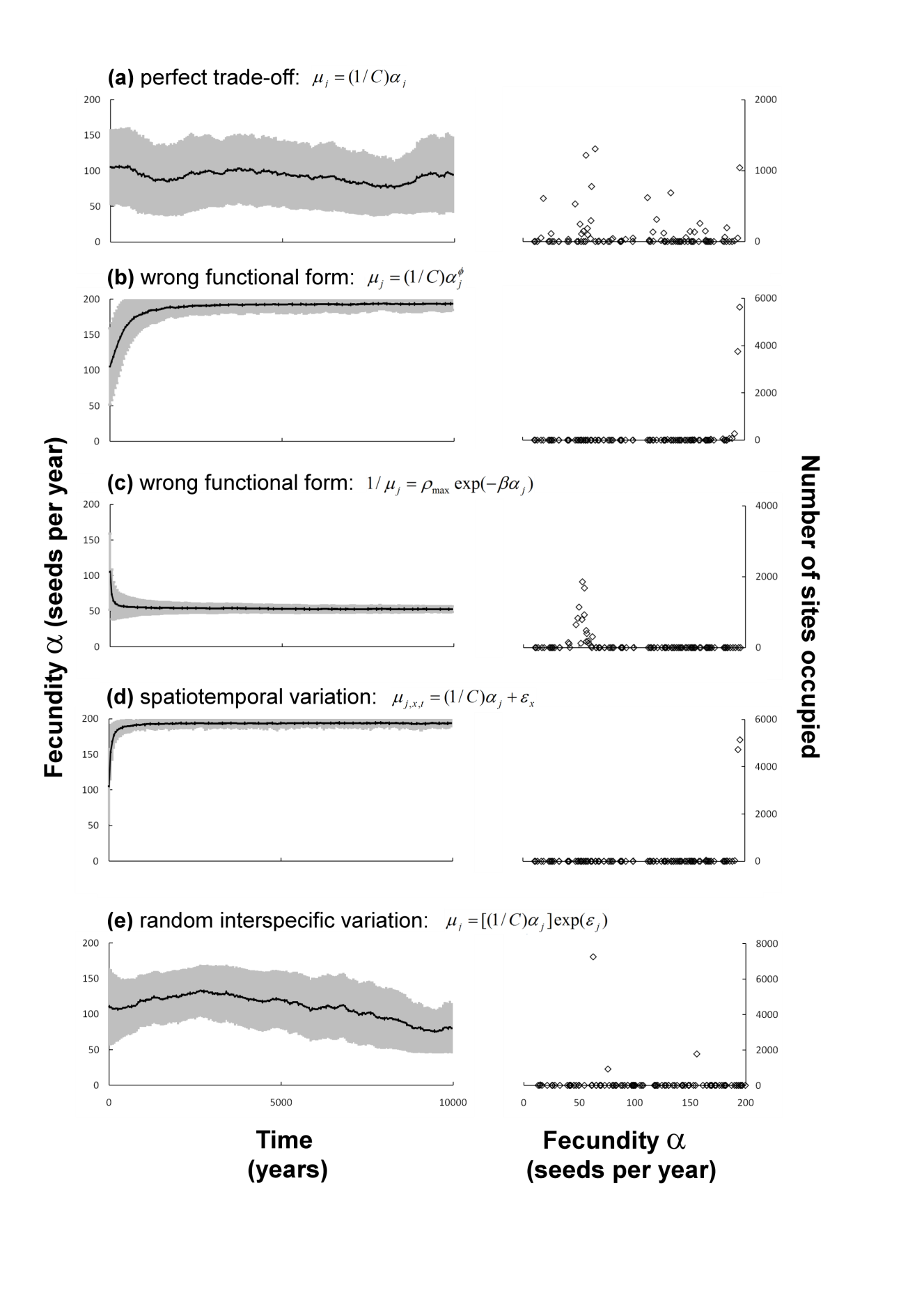
**Fig. S1**. 10,000-year simulations of space-limited communities lacking any form of niche structure or density dependence. The model used for simulations is very similar to Hubbell’s (2001) neutral model (see text). Within each community, lifespan is negatively correlated with annual fecundity according to the equation given with the panel (see main text). **Left panels**: dynamics of mean fecundity $\alpha$ (dark line: grey region gives mean ± the standard deviation). **Right panels**: state of the community at the end of the simulation, each symbol showing one species. As the simulations show, except in the special case of a perfect trade-off with no spatiotemporal variation and no random interspecific effects (a), trait diversity and / or species richness collapses (a – e). This figure is identical to Fig. 2 in the main text, except that the results in Fig. 2 come from using the original Hubbell (2001) formulation for seed dispersal, which does not allow for stochasticity in the arrival of seeds to newly vacated sites; whereas. Fig. S1 comes from using an explicit, Poisson formulation for seed arrivals (see Appendix 2).


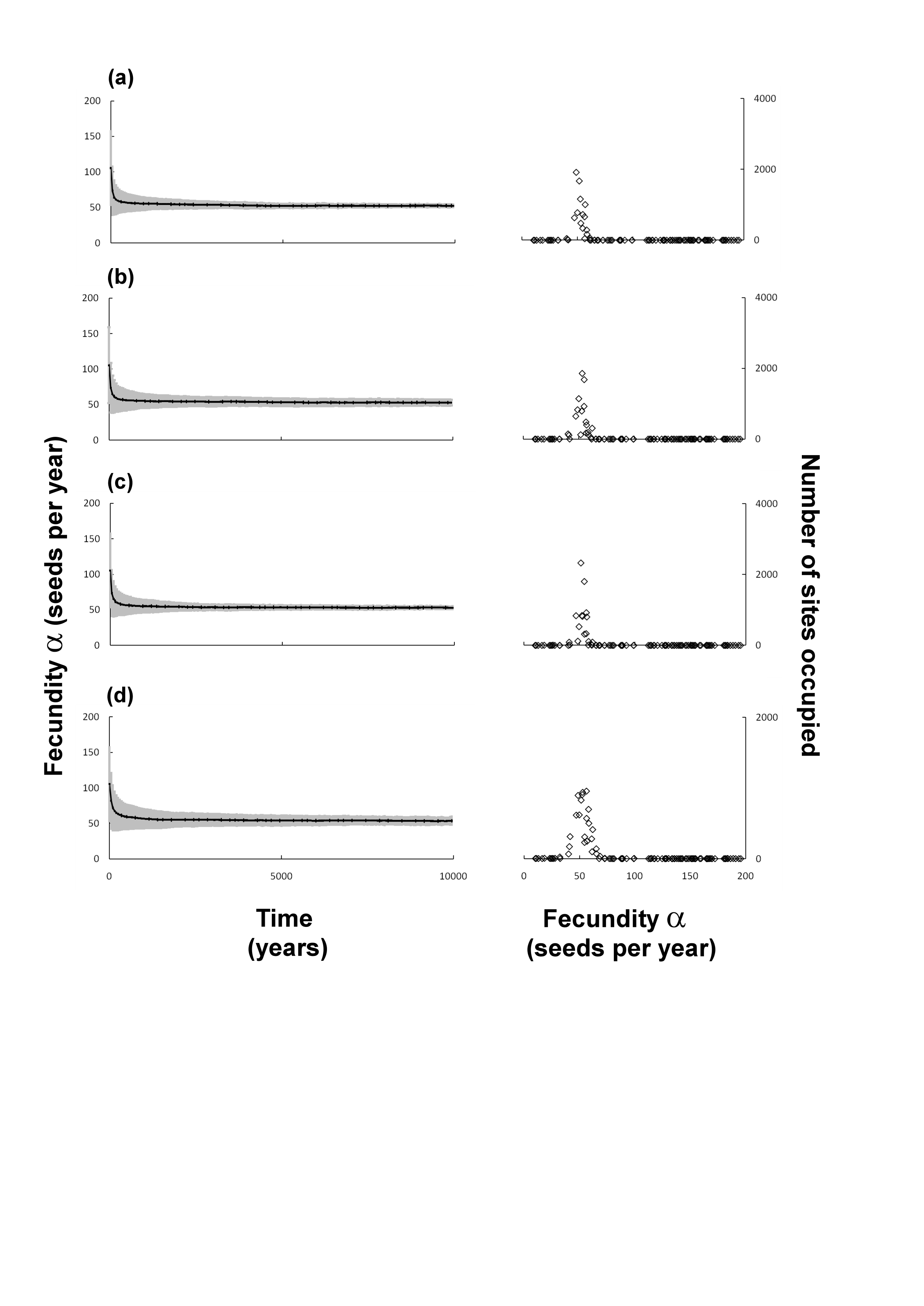
**Fig. S2.** Simulations of communities with increasing levels of stochasticity in seed arrivals. These simulations are analogous to those given in Figs 2, and S1, with an imperfect trade-off (Eq. 4). Results in (a) are from using the expectation for seed arrivals as given in Fig. 2; (b) comes stochastic seed arrivals as given in Fig. S1. (c) comes from stochastic arrivals with only a fraction 0.10 of seeds surviving; (d) comes from stochastic arrivals with only a fraction 0.010 surviving. As the results show, stochasticity of seed arrival has no material effect on the collapse of trait variation in this case.
